# Engineered marble-like bovine fat tissue for cultured meat

**DOI:** 10.1038/s42003-022-03852-5

**Published:** 2022-09-08

**Authors:** Yedidya Zagury, Iris Ianovici, Shira Landau, Neta Lavon, Shulamit Levenberg

**Affiliations:** 1 Department of Biomedical Engineering, Technion - Israel Institute of Technology Haifa, Haifa, 3200003 Israel; 2 AlephFarms Ltd., 7670609 Rehovot, Israel

**Keywords:** Tissue engineering, Stem-cell biotechnology

## Abstract

Cultured meat can provide a sustainable and more ethical alternative to conventional meat. Most of the research in this field has been focused on developing muscle tissue, as it is the main component of meat products, while very few studies address cultured fat tissue, an essential component in the human diet and determinant of meat quality, flavor, juiciness, and tenderness. Here, we engineered bovine fat tissue for cultured meat and incorporated it within engineered bovine muscle tissue. Mesenchymal stem cells (MSCs) were derived from bovine adipose tissue and exhibited the typical phenotypic profile of adipose-derived MSCs. MSC adipogenic differentiation and maturation within alginate-based three-dimensional constructs were optimized to yield a fat-rich edible engineered tissue. Subsequently, a marble-like construct, composed of engineered bovine adipose and muscle tissues, was fabricated, mimicking inter- and intra-muscular fat structures.

## Abstract (web summary)

A marbled muscle-adipose tissue construct is developed by integrating engineered bovine muscle and adipose tissue, and optimized using alginate-based 3D constructs and short differentiation times to increase adipogenic differentiation and maturation

## Introduction

Cultured meat (CM), also known as cell-based, lab-grown or cultivated meat, is created from animal cells cultured in the laboratory using tissue engineering technology^1,2^. CM can fundamentally change the global meat production marked by providing a more ethical and sustainable alternative to conventional processes. Unlike well-known meat substitutes, cultured meat steak (CMS) is developed to provide exceptional mimicry without compromising the sensory experience of conventional meat^3^. Although meat products like steaks are primarily comprised of muscle, fat presence and its lipid-composition play a crucial role in meat quality, contributing to the perception of flavors, mouthfeel sensation, appearance, texture, and nutritional value^4,5^. Fat, or adipose tissue, is formed via adipogenesis, during which mesenchymal stem cells (MSCs) differentiate into pre-adipocytes which are committed to differentiate along the adipogenic lineage. Thereafter, the cells undergo terminal differentiation, during which, they enlarge through cytosolic triglyceride accumulation and subsequently become mature adipocytes^6,7^. When generated in culture, adipogenic differentiation medium is generally comprised of standard medium supplemented with several chemical molecules^8^. However, for the production of edible tissue, it is necessary to replace these chemicals and ensure compatibility of the protocol with the food market. An adipogenic differentiation medium that contains a mixture of free-fatty acids, designed for CM production, has been developed by Mehta et al.^9^. CM production is also challenged by high production costs^3^, with culture and differentiation media expenditures comprising the main cost factor in stem cell production upscaling^10^. Thus, optimization of cell density, cell culture, and differentiation time can critically contribute to cost reduction. Moreover, to create an edible three-dimensional (3D) structure capable of supporting adipose tissue formation for CM production, the scaffolding material should be food-safe, cost-effective, and sustainable^5^. Furthermore, it should possess mechanical properties conducive to cell adipogenesis. Namely, it should exhibit low rigidity, as it is known that softer matrices enhance adipogenic differentiation level^11^, but also be capable of withstanding stress incurred during cell growth. In addition, the scaffold must easily integrate with engineered muscle tissue to form a steak-like structure.

Fabrication of structured CM composed of multiple tissue types involves complex co-culturing under co-differentiation conditions^5^. Finding suitable conditions for co-culture, especially co-differentiation, of adipose and muscle cells is challenging, since each of these cell types requires markedly different matrix properties and media composition^12^. Alternatively, following separate cultivation and differentiation, multiple tissues can be integrated, as recently shown by Kang et al. in their assembly of differentiated muscle, adipose, and blood capillary cell fibers to construct steak-like meat using 3D bioprinting^13^.

In this study, we engineered a 3D fat tissue from isolated bovine adipose-derived MSCs loaded within alginate hydrogel. Differentiation and maturation periods of the isolated MSCs were optimized to yield the most fat-rich tissue in the shortest time, under the tested conditions. To create a well-integrated structured CMS that replicates the texture of conventional meat, the engineered adipose tissue was integrated within engineered bovine muscle tissue using a gentle stitching process that allowed the co-culture of the integrated construct while preserving the delicate mature adipocytes.

## Results

### Bovine MSC isolation and characterization

Bovine MSCs (BMSCs) isolated from renal adipose tissue of an adult cattle exhibited a fibroblast‐like appearance with a spindle‐shaped morphology (Supplementary Fig. S1a). Gene expression profiling by RT-PCR analysis (Supplementary Fig. S1b) found the cells to be positive for CD29, CD73, CD105, CD90, and CD44 and negative for CD45 (Fig. S1b), a typical profile for adipose-derived MSCs^14^.

### Adipogenic differentiation of BMSCs within 3D constructs

**Fig. 1:**
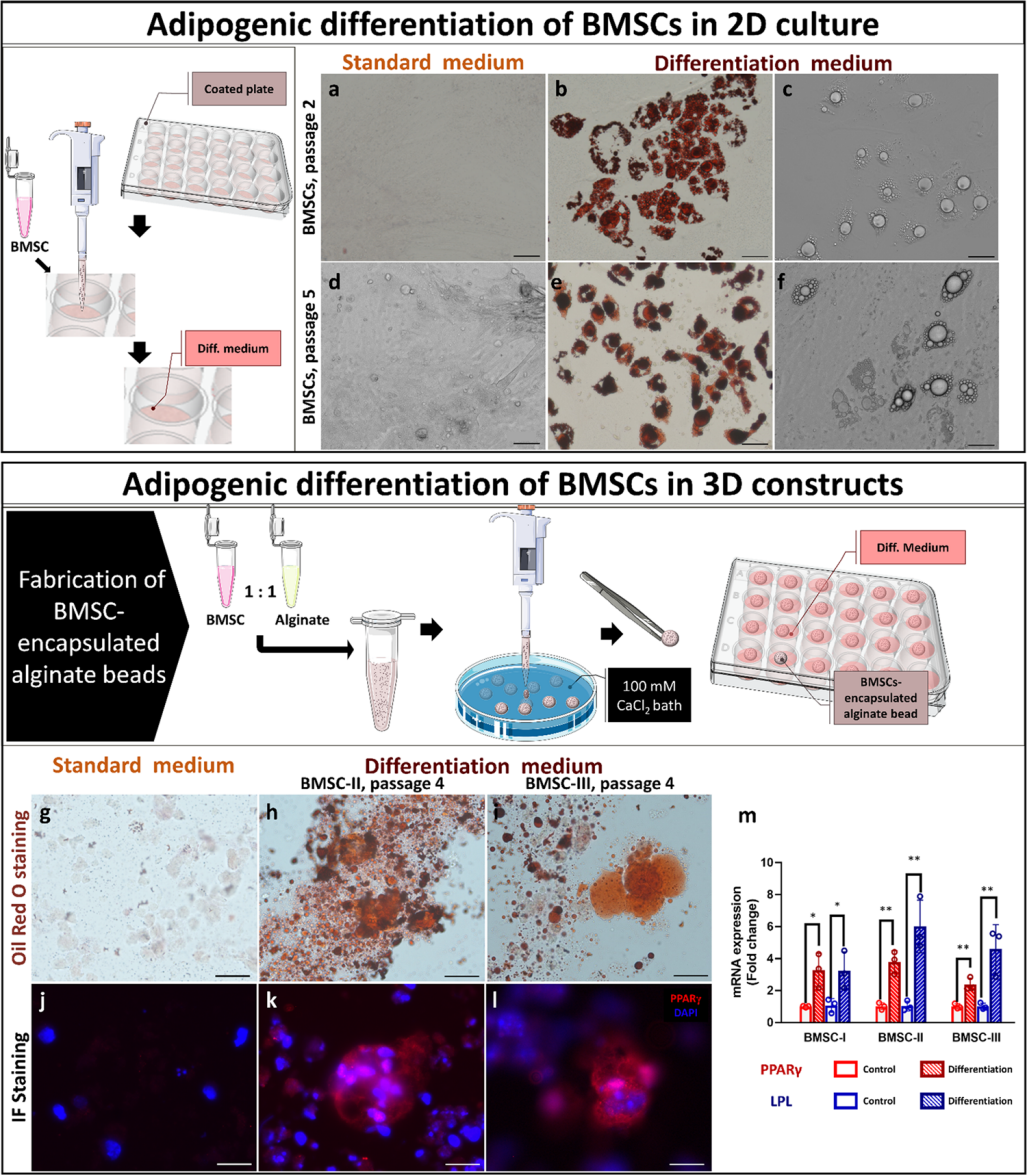
Adipogenic differentiation of isolated BMSCs within 3D construct. Top panel: BMSCs were seeded in 2D culture plates, and then differentiated into adipocytes by application of differentiation medium for 12 days. Image credits: this figure was created using elements from Servier Medical Art (https://smart.servier.com). Representative bright-field and ORO staining images of **b**, **c**, **e**, **f** post-differentiated BMSCs and **a**, **d** non-induced BMSCs at passages 2 and 5; scale bar 50 µm. Bottom panel: BMSCs were loaded into 0.5% alginate hydrogel beads and cultured in differentiation medium (illustration of the process in the bottom panel). Image credits: this figure was created using elements from Servier Medical Art (https://smart.servier.com). ORO-stained sections of BMSC-loaded beads cultured in **g** standard or **h**, **i** differentiation medium for 19 days. PPARγ-stained sections of BMSC-loaded beads cultured in **j** standard or **k**, **l** differentiation medium for 19 days (red, PPARγ; blue, DAPI); scale bar 20 µm. **m** Relative fold-change of LPL and PPARγ mRNA expression, measured using real-time qPCR, in differentiated BMSCs of each isolation (BMSC-I, BMSC-II, and BMSC-III). Fold-change in expression was calculated using the ΔΔCt method after normalization with endogenous reference 18s or GAPDH. Differences between gene expression levels of BMSCs loaded into beads cultured in differentiation versus standard medium (control) were evaluated using Student’s *t*-test and reported as the mean ± SE of triplicates, *p < 0.05, **p < 0.01.

Towards the goal of bioengineering a 3D adipose tissue-like construct composed of isolated BMSCs, the isolated cells were first induced to differentiate into adipocytes in 2D culture. At passages 2–5, the BMSCs adipogenic differentiation was triggered by application of differentiation medium for 12 days. Twelve days post-differentiation, BMSC had accumulated lipids in their cytosol and acquired a round shape, typical of mature adipocytes (Fig. 1 top panel; b, c, e, f). The adipogenic differentiation was confirmed by Oil Red O (ORO) staining (Fig. 1b, e).

After establishing their adipogenic differentiation capacities in 2D culture, BMSCs were loaded into 3D alginate hydrogel beads and were subjected to the same differentiation conditions. Lipid droplet accumulation, as visualized by ORO staining and peroxisome proliferator-activated receptor gamma (PPARγ) staining, indicated robust differentiation of BMSCs upon adipogenic stimulation (Fig. 1, bottom panel). In parallel, lipoprotein lipase (*LPL*) and *PPARγ* expression levels were 3–6 times higher than in non-induced BMSCs cultured in standard medium only (Fig. 1m).

### Influence of matrix material on adipose tissue formation

**Fig. 2:**
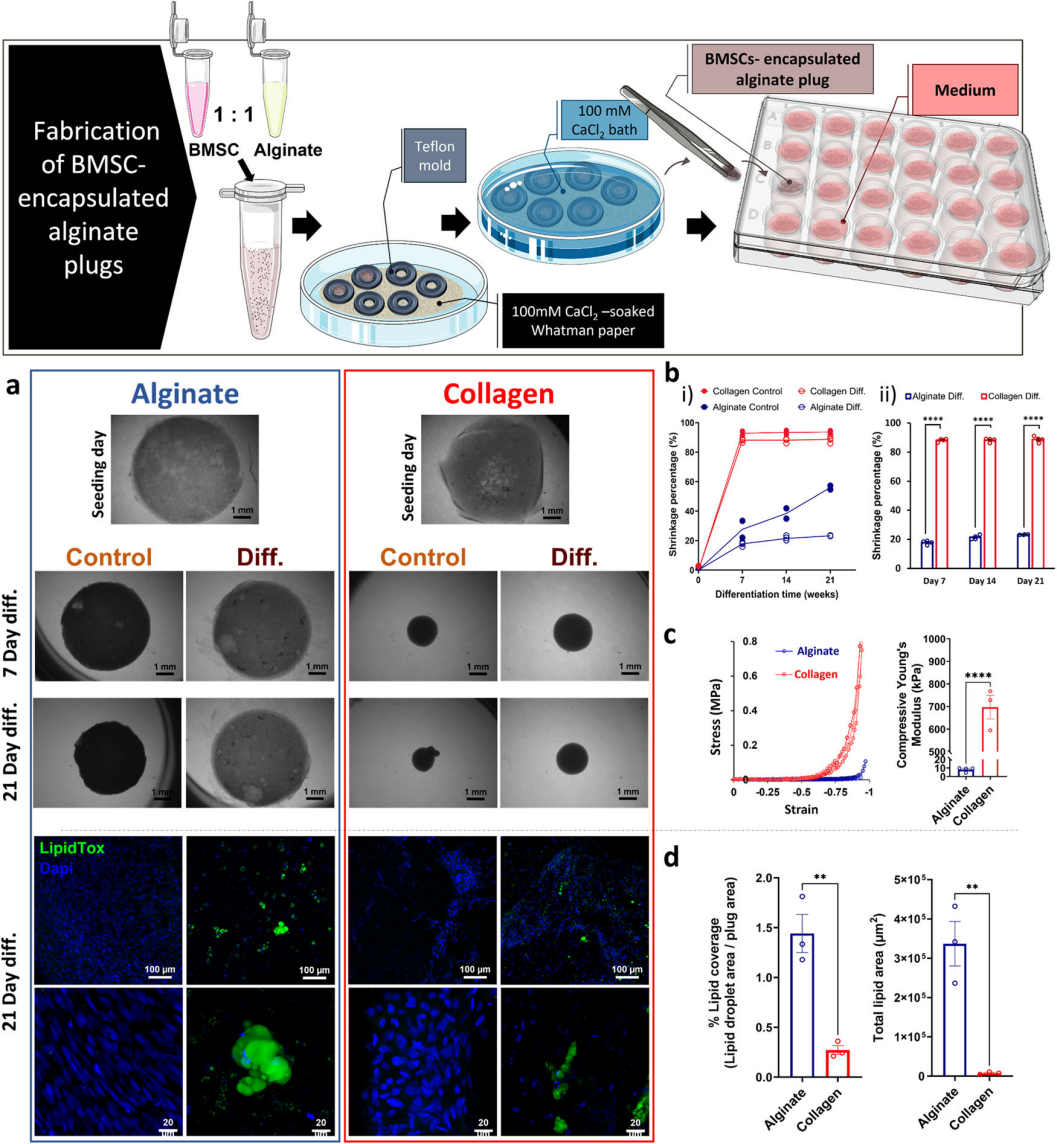
Influence of matrix material on engineered adipose tissue formation. Top panel: Illustration of BMSC-loaded alginate plug fabrication. Image credits: this figure was created using elements from Servier Medical Art (https://smart.servier.com). **a** (top panel) Bright-field images of BMSC-loaded alginate and BMSC-loaded collagen constructs cultured in differentiation medium (diff.) or standard medium (control), as captured on seeding day, and after 7, 14, and 21 days of differentiation, scale bar 1 mm. **b** Shrinkage percentage of the constructs (relative to their original size) (i) All samples: differentiation or control in alginate or in collagen over 21 days of differentiation, (ii) BMSC-loaded alginate vs. BMSC-loaded collagen constructs after 7, 14, and 21 days in differentiation medium *n* =4. **c** Compressive stress–strain curves and calculated Young’s modulus of BMSC-loaded alginate vs. BMSC-loaded collagen constructs after 21 days in differentiation medium, *n* = 3, Student’s *t*-test, error bars show SE, ****p < 0.0001. **a** (bottom panel) Confocal laser scanning microscopy images (green, LipidTox; blue, DAPI) of BMSC-loaded alginate and BMSC-loaded collagen constructs cultured in differentiation medium or in standard medium, for 21 days, scale bars: 100 µm (top) and 20 µm (bottom). **d** Quantified lipid droplet coverage and total lipid area of LipidTox-stained differentiated alginate vs. collagen constructs, *n* = 3, Student’s *t*-test, error bars show SE, **p < 0.01.

To assess the effect of matrix composition on adipogenic differentiation, BMSCs were suspended in 0.5% alginate or in 0.5% collagen-I to form 3D BMSC-loaded plug constructs. In this technique, 1-mm-thick 3D hydrogel plugs were generated using a casting technique. BMSC-loaded collagen constructs incubated in either differentiation or standard medium shrank significantly (around 90% relative of their original size), while the BMSC-loaded alginate constructs in differentiation medium retained their shape and shrank by about only 20% after 21 days of differentiation (Fig. 2b). In contrast, after 21 days of differentiation alginate constructs cultured in standard medium were 50% their initial size, likely due to massive cell proliferation, wherein, the high cell density caused partial contraction of the construct (Fig. 2b). BMSC-loaded alginate constructs were significantly softer when compared to BMSC-loaded collagen constructs (Young’s modulus 8 ± 1 kPa versus 697 ± 52 kPa, respectively) (Fig. 2c). An enlarged graph of the linear region of the stress–strain curves and their linear regression trend lines, through which the slopes (i.e., the Young’s modulus) were calculated is displayed in supplementary Fig. S2. In addition, the lipid droplet coverage and total lipid droplet count were significantly higher in BMSC-loaded alginate versus BMSC-loaded collagen constructs (Fig. 2d). Taken together, alginate as compared to collagen is a superior matrix for the formation of 3D adipose tissue.

### Optimizing adipogenic differentiation and maturation periods

**Fig. 3:**
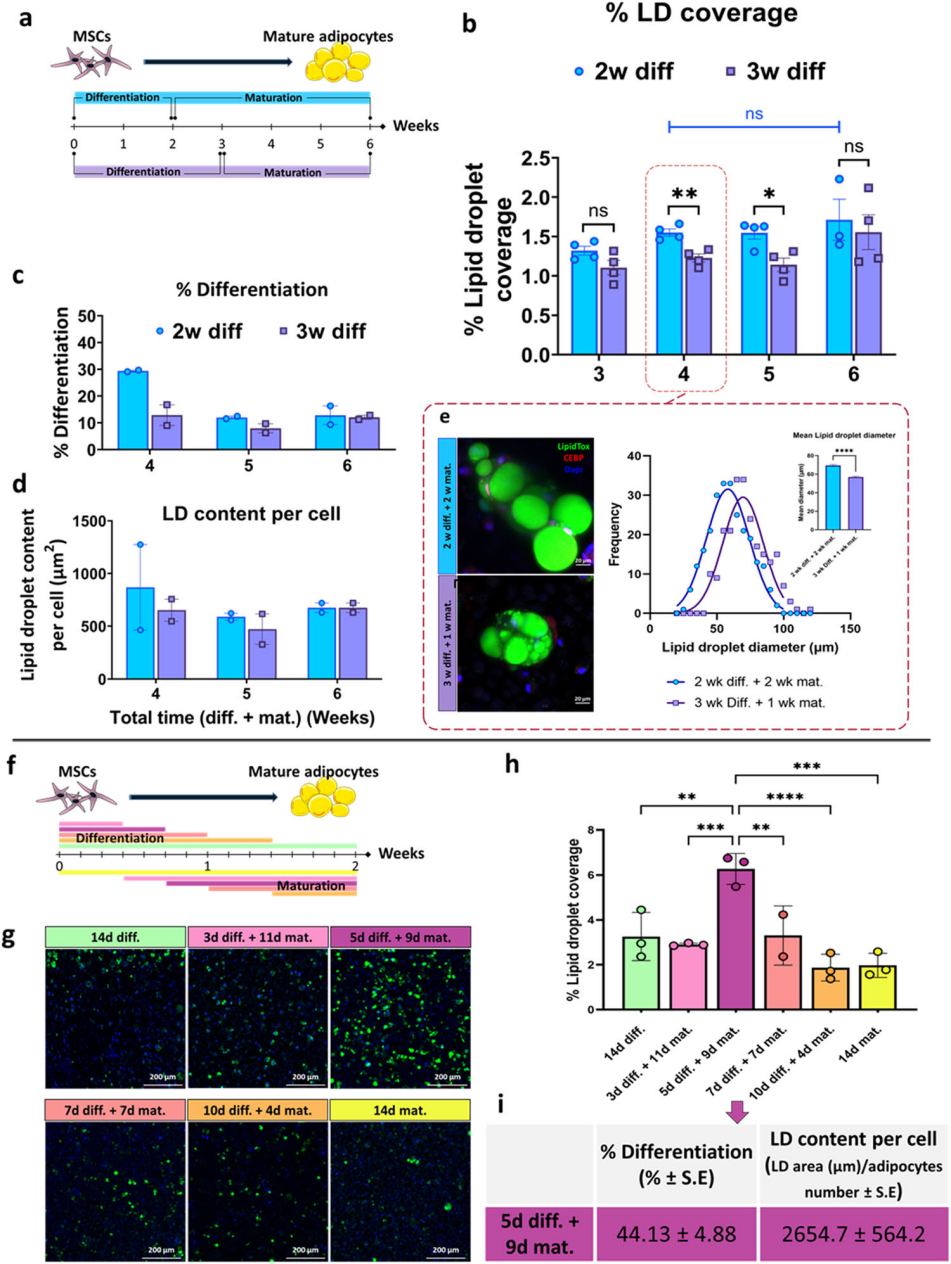
Optimization of adipogenic differentiation and maturation periods of BMSC-loaded alginate 3D constructs. **a** Scheme of the 6-week experimental timeline. Image credits: this figure was created using elements from Servier Medical Art (https://smart.servier.com*)*. **b** Percentage of lipid droplet coverage, **c** differentiation percentage, and **d** lipid droplet content per differentiated cell, in BMSCs subjected to 2 weeks of differentiation followed by 1–4 weeks of maturation (blue circles) or to 3 weeks of differentiation followed by 2–3 weeks of maturation (purple squares); *n* = 4. Multiple unpaired *t*-test, Holm-Sidak post hoc, error bars show standard error (SE), ns: non-significant, *p < 0.05, **p < 0.01. **e** Whole-mount C/EBPα and lipid staining (red, C/EBPα; green, LipidTox; blue, DAPI), lipid droplet size distribution and mean diameter after 2 weeks of differentiation followed by 2 weeks of maturation (blue, ‘2w diff. + 2w mat.’) and after 3 weeks of differentiation followed by 1 week of maturation (purple, ‘3w diff. + 1w mat.’); Student’s *t*-test, error bars show SE, ****p < 0.0001. **f** Scheme of the 2-week experimental timeline, each color line represents a different experimental timeline. **g** Whole-mount lipid staining (green, LipidTox; blue, DAPI) and **h** lipid droplet coverage percentage of lipid-stained constructs subjected to different differentiation/maturation schedules, *n* = 4, one-way ANOVA followed by Tukey’s post hoc test, **p < 0.01, ***p < 0.001, ****p < 0.0001, error bars show SE. **i** Mean (±SE) differentiation percentage and lipid droplet content per cell in lipid-stained BMSC-loaded constructs after 5 days in differentiation medium followed by 9 days in maturation medium, *n* = 3. Each color represents unique combination of differentiation and maturation periods as specified in the figure; d = days; diff. = differentiation medium; mat. = maturation medium.

After selecting the matrix material for adipogenic differentiation in 3D, the differentiation profile of BMSCs in 3D alginate constructs was evaluated over time (Fig. S3a). Whole-mount immunofluorescence staining for CCAAT/enhancer-binding protein alpha (C/EBPα) (an adipogenic marker), and LipidTox revealed larger adipocytes with higher lipid droplet content in their cytosol after 21 days of differentiation as compared to 14 days (Fig. S3b). To obtain more mature adipocytes and thereby enrich the construct with lipid droplets, a maturation phase was added to the construct preparation protocol, using two methods: (i) short differentiation—2 weeks of differentiation and then 1–4 weeks of maturation, or (ii) long differentiation—3 weeks of differentiation and then 1–3 weeks of maturation, totaling a maximum 6-week differentiation and maturation period in both cases (Fig. 3a–e). To compare the outcomes of each protocol, the percentage of lipid droplet coverage, differentiation percentage and lipid droplet content per differentiated cell were quantified (Fig. 3b–d). At the 4-week time point, the lipid droplet coverage was significantly higher using the first method as compared to the second method (Fig. 3b). Within each method, when comparing between 4-, 5-, and 6-week time points, the increase in the lipid droplet coverage was not statistically significant (Fig. 3b). In addition, at the 4-week time point, larger fat-laden adipocytes accumulated in constructs with the short differentiation (2 weeks), as indicated by LipidTox staining and by the lipid droplet size distribution analysis, which showed a significantly higher average diameter (Fig. 3e). In conclusion, 2 weeks of differentiation followed by 2 weeks of maturation provided for the richest 3D engineered bovine adipose tissue in the shortest time, under the tested conditions.

To further optimize culture conditions toward the goal of cost-effective production of CM, the impact of shortening the adipogenesis period and decreasing cell density were evaluated. To this end, cells were seeded at a lower density and subjected to a 2-week adipogenesis protocol, comprised of differentiation and maturation phases of different lengths (Fig. 3f–i). The highest lipid content was achieved after 5 days of differentiation followed by 9 days of maturation (Fig. 3g, h). The differentiation percentage (i.e., the percentage of cells that had undergone adipogenic differentiation) and lipid droplet content per cell was significantly higher compared to the 4–6-week protocol (differentiation: ~45% vs. 10–30%, respectively; mean lipid droplet content per cell: ~2700 µm^2^ vs. 500–1000 µm^2^, respectively (Fig. 3c, d, i)).

### Engineered bovine adipose-muscle marbled-like construct

To mimic the structure of a natural steak, a combined fat and muscle 3D construct was created mimicking inter- and intra-muscular fat.

### Mold-cast marbled-like construct

**Fig. 4:**
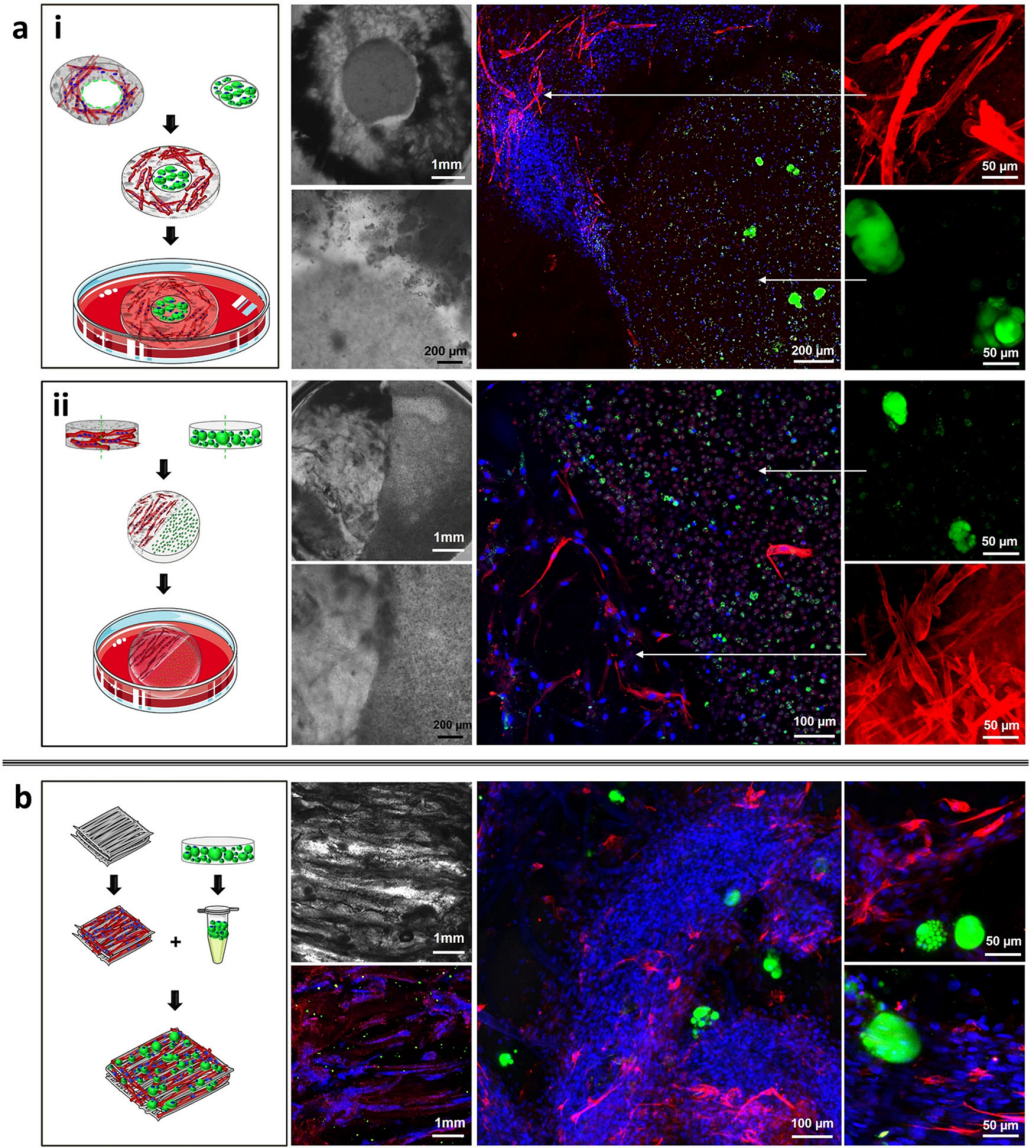
Engineered bovine adipose-muscle marbled-like construct. **a** Engineered mold-cast marble-like constructs composed of bovine fat and muscle in a (i) ring and a (ii) semicircular configuration. The outer ring/left semi-circle is a lyophilized casted alginate-RGD plug onto which bovine satellite cells were seeded and myogenically differentiated into myotubes. The inner ring/right semi-circle is an alginate plug loaded with differentiated BMSCs. The integrated constructs were incubated in adipogenic maturation medium for 1 week. From left to right: Illustration of the attachment process, bright field images in low and high magnifications, and confocal images of lipid and desmin staining of constructs (green, LipidTox; red, Desmin; blue, DAPI) in low and high magnifications. **b** Marbled-like construct on a 3D-printed scaffold composed of extracted mature adipocytes (differentiated BMSCs) within bovine engineered muscle tissue. From left to right: illustration of the construct fabrication process, bright field image, and confocal images of lipid and desmin staining of constructs (green, LipidTox; red, Desmin; blue, DAPI) at different magnifications. Image credits: this figure was created using elements from Servier Medical Art (https://smart.servier.com).

To mimic intermuscular fat structure, mature engineered adipose tissue, formed of BMSCs and mature engineered muscle tissue, formed of bovine satellite cells (BSCs), were cultured separately for up to 1 month. Then, the engineered adipose and muscle constructs were integrated in a ring shape (Fig. 4 a(i)) and in a semicircular shape (Fig. 4 a(ii)). This was achieved by localized chelation of calcium ions at the connection area and re-cross-linking of the alginate using calcium solution. The combined tissues were co-cultured in adipogenic maturation medium for 1 week to achieve a fully integrated marble-like construct (Fig. 4a). The mold-cast marble-like constructs appeared to be integrated, without detachment points after 1 week of co-culture (Fig. 4a and Fig. S4a). Desmin-stained myotubes alongside mature adipocytes took on the ring and semicircular configurations of the mold (Fig. 4a). To further prove the differentiation of the satellite cells into muscle cells, confocal laser scanning microscope (LSM) images of samples stained for myogenin, a muscle-specific transcription factor involved in myogenesis, were taken from the areas of the muscle in the integrated constructs (Supplementary, Fig. S4b).

### Marble-like construct on 3D-printed scaffold

To mimic the structure of intramuscular fat, we developed a technique for extracting the mature adipocytes from the 3D alginate construct and incorporated them within an engineered muscle tissue which was made via 3D-printing (Fig. 4b). Staining of lipid and desmin demonstrated that the mature adipocytes remained intact, retained their lipid droplets during the extraction process, and were surrounded by muscle fibers, possessing a marble pattern, like marbled meat (Fig. 4b).

**Fig. 5:**
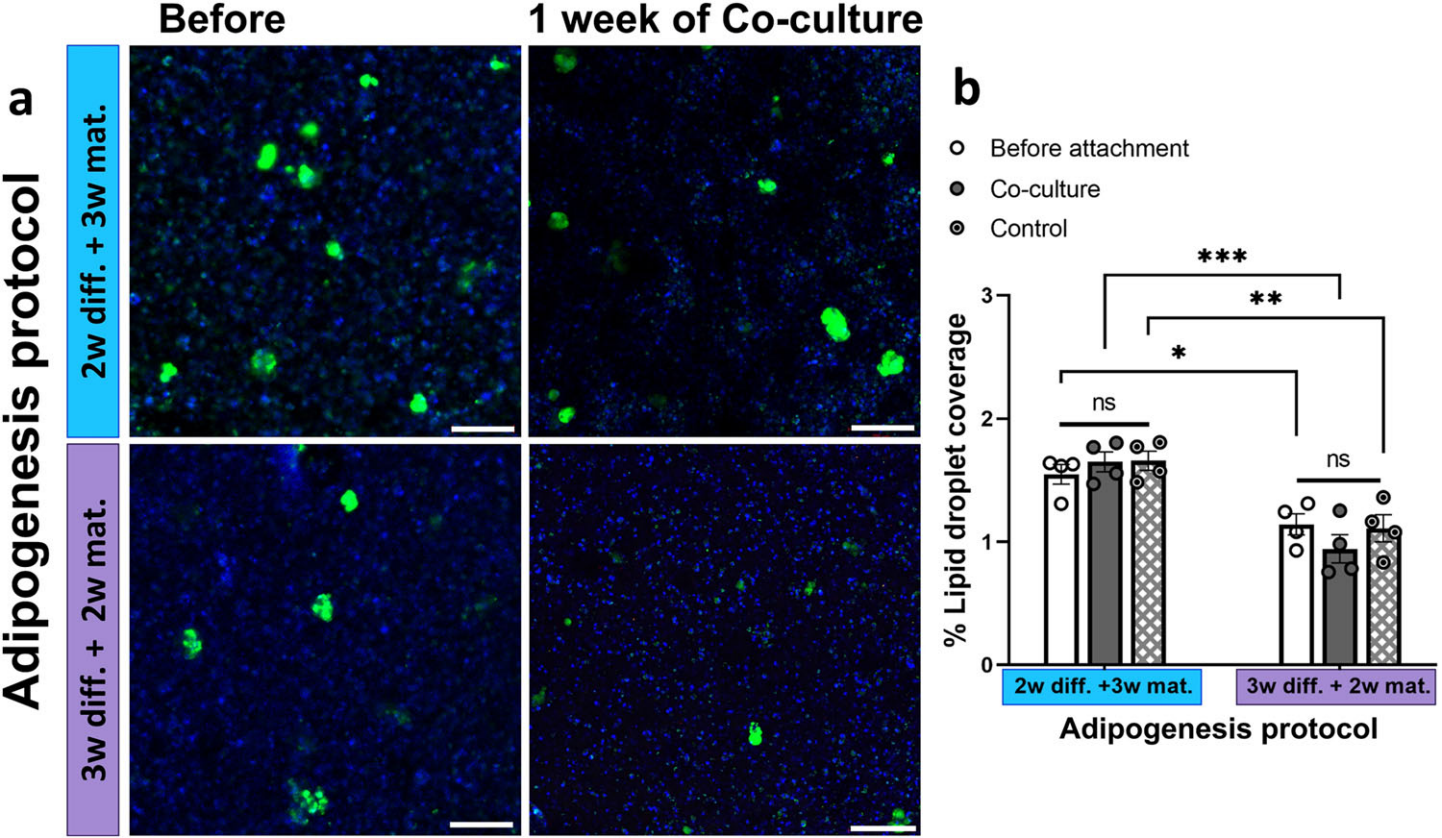
Comparative analysis of mature adipocytes within marble-like constructs. **a** Confocal images of lipid staining of the ring-shape integrated constructs before the integration procedure versus after integration and 1-week co-culture, for 2 types of adipose constructs; 2-week differentiation followed by 3-week maturation (‘2w diff. + 3w mat.’) and 3-weeks differentiation followed by 2-week maturation (‘3w diff. + 2w mat.’). The images were taken from the inner ring region of the integrated constructs (green, LipidTox; blue, DAPI), scale bar 200 µm. **b** Lipid droplet coverage percentage of the Lipid-stained constructs before the integration procedure ‘Before attachment’, after the integration procedure and 1-week co-culture in maturation medium (‘Co-culture’), and of constructs that contained only adipocytes (adipose construct that were integrated with non-cell scaffold) after the integration procedure and 1-week co-culture in maturation medium (‘Control’) for the ‘2w diff. + 3w mat.’ and ‘3w diff. + 2w mat.’ adipose constructs. *n* = 4, one-way ANOVA followed by Tukey’s post hoc test, ns: non-significant, error bars show standard error.

To further evaluate the potential of co-culturing the integrated adipose-muscle construct, a quantitative comparison of constructs before the integration procedure versus after integration and co-culture, for 2 types of adipose constructs (i.e., that were generated by different adipogenesis protocol) was conducted (Fig. 5). For this experiment, BMSCs-loaded alginate plugs were subjected to ‘2w diff. + 3w mat.’ or the ‘3w diff. + 2w mat.’ adipogenesis protocol. Then, the constructs were integrated with muscle cells-loaded alginate plugs or cell-free alginate plugs (‘control’), in a ring-shape configuration, and were co-cultured for 1 week. The integrity of the mature adipocytes, which are known to be highly sensitive, as a result of the integration procedure and throughout 1 week of co-culture was assessed by measuring the lipid content (Fig. 5). Lipid content was preserved following the integration process and the 1-week co-culture period in both adipose construct types (i.e., ‘2w diff. + 3w mat.’ and ‘3w diff. + 2w mat.’) (Fig. 5a). Furthermore, the lipid content was not affected by muscle cell presence, as determined by comparing the lipid droplet content in the constructs containing both adipocytes and muscle cells (‘co-culture’; Fig. 5b) to those containing adipocytes only (‘control’; Fig. 5b).

## Discussion

In this study, we engineered edible fat tissue for cultured steak production. The fat-rich 3D construct was created by loading an alginate hydrogel with isolated adipose-derived BMSCs, and allowing them to differentiate into mature adipocytes. In addition, we optimized differentiation and maturation periods to achieve lipid-rich tissue within minimal culture time. To create an edible marble-like construct, we then integrated engineered bovine fat tissue and muscle tissue using two techniques: (1) a gentle attachment technique that allows for continued co-culture; (2) extraction of mature adipocytes, that were cultured in alginate hydrogel, and placing them within the muscle tissue. The pioneering CM product first produced in 2013, was composed of muscle cells only^15^, as opposed to natural steak which consists of varying proportions of both muscle and fat. The fat melts during cooking and lubricates the muscle fibers, thus providing juiciness and mouthfeel.

CM production requires a reliable and reproducible stem cell source. Bovine adipose-derived MSCs are promising adult stem cells due to their multiple potential extraction sites, easy isolation, and high proliferation capacity in vitro^16^. In the present work, the cells from three independent extractions were characterized for their MSC morphology and gene expression and demonstrated a profile typical of adipose-derived MSCs^14^. The independent isolations produced consistent results in terms of morphology and gene expression, indicating successful and reproducible BMSCs isolation protocol.

CM production requires extensive cell expansion prior to the differentiation phase, to yield high volumes of cell material from a limited donor tissue. Repetitive passaging, however, may come at the cost of loss of differentiation potential. Indeed, the adipogenic potential of human MSCs has been shown to decline with increasing passages^17,18^, alongside increased adipogenic potential with older donor age^18^. In contrast, bovine adipose-derived MSCs showed significantly lower adipogenic differentiation in passage 5 as compared to passage 2^19^. However, here we present preserved BMSC adipogenic differentiation capacity throughout passages 2–5. Further research will be needed to explore the limits of BMSC expansion and adipogenic differentiation capacity.

CM production involves tissue engineering techniques typically applied for regenerative medicine, and requires several adaptations such as mass upscaling, economically efficient production, and replacement of animal-derived and non-edible materials used for cell growth and scaffolding, with suitable substitutes. Future CM developments will have to address all of these points to ensure its integration in the conventional meat market. Furthermore, the cell requirements must be kept in mind. Specifically, to create adipose tissue, it is essential to grow and differentiate the cells within a 3D structure with stiffness, adhesivity, and porosity suitable for adipogenesis induction. Human MSCs have been shown to differentiate into adipocytes on wide range of 3D scaffold matrices such as PCL nano-fibrous^20^, PET fibrous^21,22^, silk^23^, and others. However, these materials are oriented for biomedical applications and are not suitable for food applications. While natural biopolymers like fibrin, hyaluronic acid, and chitosan are likely food-safe, their use in CM production is limited because they are animal-derived materials and although they can be recombinantly produced, the process may be cost-inefficient for CM. Moreover, adipogenic induction requires specific mechanical and physical properties. Therefore, finding the optimal scaffold candidates for adipose tissue formation for CM applications is challenging. In the current study, we tested two different scaffold materials and fabrication techniques. Collagen was selected as the scaffold, as it is known to support MSC adhesion and has been previously used as scaffolding material to generate adipose tissue from human MSCs^24^ and bovine adipose-derived MSCs^13^. Also gelatin, a natural polypeptide derived from partial denatured collagen, has been applied to engineer adipose tissue from human MSCs^25^. In the present work, alginate was chosen as it was previously shown to support adipogenic differentiation of bovine MSCs^9^ and human MSCs^26,27^. Both scaffolds are edible and can be produced as recombinant or plant-based forms. Alginate enabled more robust adipogenic differentiation compared to collagen. Moreover, unlike alginate, collagen constructs underwent significant shrinkage which most likely driven by the contractile forces of the cells, which resulted in higher construct stiffness. The mechanical properties of cell matrices are known to influence MSC fate. Xie et al. showed that stiffer substrates activated β-catenin signaling, which triggered osteogenic differentiation, whereas soft substrates inhibited β-catenin while increasing PPARγ expression, consequently enhancing adipogenesis^28^. According to our results, the stiffness of alginate constructs, as measured by compression test, after cell differentiation was comparable to the stiffness of other substrates which were demonstrated to support adipogenesis^28,29^. In addition to stiffness, meat culturing materials must maintain their original dimensions throughout growth and differentiation. Other requirements of a scaffold for CM are accessibility and affordable cost. Due to the fact that alginate is abundantly available in nature and significantly less expensive than recombinant collagen and based on our observations, we selected alginate as a substrate for further experiments.

The ultimate goal of edible tissue engineering is to create tissue that will best mimic real fat tissue, i.e., lipid-rich adipose tissue, in the shortest culture time. Understanding the natural process of adipogenesis may contribute to reaching this goal. At the cellular level, adipogenesis involves three main stages: (1) commitment of MSCs to the adipocyte lineage, (2) expansion of the committed cells, and (3) terminal differentiation, which is associated with a dramatic increase in lipogenesis. Each stage, particularly the differentiation of pre-adipocytes into mature adipocytes, is influenced by specific growth factors and adipogenic hormones, which control cell differentiation, growth, and cell fate specification. For instance, some factors stimulate proliferation of pre-adipocytes but inhibit differentiation of pre-adipocytes into mature adipocytes^30^. Therefore, in order to achieve optimal differentiation and fat accumulation within the cells, growth, differentiation and maturation time, cell density and other parameters should be optimized. Here, we evaluated lipid coverage as a function of differentiation time and optimized the ratio between differentiation and maturation periods. Previous studies have shown that addition of a maturation phase enhances adipocytes preservation and increases lipid storage in the cytosol^31–34^. In this work, the richest 3D engineered bovine adipose tissue was obtained after subjecting BMSC-loaded scaffolds to a 2-week differentiation and 2-week maturation regimen. In this combination, the size and amount of lipid droplets were significantly higher than after other tested schedules, indicating an advanced maturity of the embedded adipocytes. It is known that the average size of mature animal adipocytes is a function of lipid accumulation within the cytosol, and is affected by species, gender, age, and diet, among other factors^27^. For example, adipose tissue of salmon fish is characterized by relatively small adipocytes (<50 µm diameter) compared to “high-marbled” muscle-fat tissue of cattle that is characterized by large adipocytes (>100 µm diameter)^27^. Therefore, when engineering adipose tissue for CM, we sought to create large adipocytes to more accurately mimic the natural structure of bovine fat, as was successfully obtained.

Further improvements of culture conditions were investigated, resulting in significant enhancement of differentiation percentage and lipid droplet content while shortening culture time. These improvements will directly reduce production costs^35^. The end-purpose of generating edible fat tissue is to integrate it within muscle tissue to create CMS. One of the challenges in developing a multi-tissue-type construct is to find the optimal culture conditions that will support selective differentiation of multiple stem cell types. This challenge can be overcome using a two-step process, as reported by Strobel et al.^31^. This approach first involves cell commitment to a specific lineage, usually the sensitive cell type that does not differentiate spontaneously, before combining it with another cell type and co-culturing them in an optimized medium. Alternatively, Shahin-Shamsabadi et al. combined two types of sheets in parallel, made of partially differentiated cells, to create a multi-tissue type construct^36^. However, co-culture of adipocytes and muscle cell precursors have been shown to suppress muscle cell differentiation^37–39^ and inhibit adipogenic differentiation^40^. A third approach grows and fully differentiates each cell type separately and then combines them to form an integrated construct. This approach can be applied for CM production and may be the most feasible and cost-effective among the mentioned approaches. Several recent publications have reported on application of this approach for structured CM development^13,36^. Although Shahin-Shamsabadi et al. attempted to create a multi-tissue-type construct for CM, they used model cell lines (murine C2C12 and 3T3-L1), which may not adequately represent primary cells^36^. The third approach involves attachment technique of the separated-grown tissues, while Kang et al. used transglutaminase over two days at 4 °C^13^, which terminates cell growth, the present work used a gentle attachment technique that preserve the cell viability and enable further co-culture of the integrated multi-tissue construct. In the current study, separately grown, differentiated and fully mature tissues formed of BMSCs and BSCs were integrated to form a marbled-like construct. The mold-casted construct was prepared in two geometrical orientations, mimicking intermuscular fat structure with mature adipocytes located between individual muscles. The marble-like construct fabricated on 3D-printed scaffold, mimicked intramuscular fat structures, which contribute to meat juiciness, flavor, and tenderness^41^. The integrated mold-casted construct was co-cultured in adipogenic maturation medium for 1 week following the attachment procedure. It is important to note that the purpose of this experiment was to assess the potential of attaching two mature tissues while preserving the cells in each of the tissues during a gentle stitching process and throughout the co-culturing. The 1-week co-culture step was implemented in attempt to generate a smooth and well-integrated co-construct, which will likely contribute to the feeling of whole meat-like cut that will not disintegrate during processing/heating.

Preparation of the marble-like construct on 3D-printed scaffold reported here involved an innovative technique of extracting the mature adipocytes from the alginate hydrogel while maintaining their integrity, loading them in alginate solution and cross-linking it on the engineered muscle tissue. This technique may open doors for a variety of applications. It is possible to control the concentration of the extracted mature adipocytes (it can be concentrated or diluted). They can be further loaded within a desired biomaterial, not necessarily the one required to induce their differentiation, thus the final scaffold material of the product can be replaced in this stage. Under appropriate conditions, the extracted mature adipocytes can be 3D bio-printed to form “highly structured” meat products^42^.

Both attachment techniques tested here yielded intact and physically stable combined constructs containing bovine fat and muscle cells. Neither the attachment process nor the 1-week co-culturing phase impaired adipocyte integrity. In addition, the presence of the engineered muscle tissue did not impair the mature adipocytes after one week of co-culture. These findings encourage and reveal an additional interesting and instructive information in the complexity of co-culture systems, however, it is certainly worthy of further investigation.

Overall, this study sought to engineer bovine fat tissue for cultured meat by encapsulating adipose-derived bovine MSCs within a 3D alginate scaffold and differentiating them into mature adipocytes. As described herein, efforts were made to economically optimize the production process by reducing culture times. Several strategies may improve CM production such as increasing cell growth and differentiation efficiency or reducing the costs of media for example by substituting low-cost growth factors. Moreover, evaluating the organoleptic properties of the cultured construct, as well as the fatty acid profile of the engineered adipose tissue, is essential for the development of cultured meat and should be further investigated in future studies.

This work reported on engineering of bovine fat tissue for cultured meat which entailed isolation of adipose-derived bovine MSCs, their encapsulation within a 3D alginate hydrogel and their later differentiation into mature adipocytes. Various matrices, fabrication techniques, and differentiation and maturation schedules, were evaluated to optimize the production of fat tissue for cultured meat. BMSCs in a 3D alginate matrix exhibited more robust adipogenic differentiation, while maintaining the construct dimensions, compared to those grown on a collagen matrix. The optimized process yielded lipid-rich constructs within only 2 weeks. Subsequently, we established two techniques for integration of engineered bovine fat tissue within engineered bovine muscle tissue, to imitate inter- and intra-muscular fat structures toward the goal of creating cultured steak.

## Methods

### Adipose-derived stem cell isolation and expansion

BMSC were isolated from the peri-renal adipose tissue of a 1-year-old Holstein Friesian cattle carcass. The resected tissues were soaked immediately in sterile phosphate-buffered saline (PBS) supplemented with 3% penicillin-streptomycin-nystatin solution (PSN; Biological Industries). The tissues were washed 3× with PBS/PSN, cleaned of residual blood vessels and connective tissue and minced with a sterile tweezer/scissors on a plastic dish. Then the tissue was digested with collagenase (type 1; Gibco) diluted in Dulbecco’s modified Eagle medium (DMEM; Gibco). Collagenase activity was terminated by addition of an equal volume of DMEM. The digested tissue was then passed through a 100-μm mesh filter and centrifuged at 1500 × *g* for 10 min at room temperature. The supernatant was discarded, and the pellet was resuspended in BMSC medium (DMEM - high glucose (DMEM-HG; Gibco) supplemented with 10% fetal bovine serum (FBS; Hyclone). This initial phase of the primary cell culture was identified as passage 0 (P0). Cells were cultured in an incubator at 37 °C under 5% CO_2_. After 4 days of culture, cells were washed twice with PBS to remove unattached cells and cultured in fresh medium. At 80% confluence, the cells were harvested with 0.25% trypsin-EDTA, reseeded at a density of 5 × 10^3^ cells/cm^2^ (P1) and maintained in BMSC medium.

### Cell-loaded 3D hydrogel constructs

#### BMSC-seeded alginate matrix

BMSCs were cultured until 80–85% confluent, detached with trypsin-EDTA, and suspended in BMSC medium. The cell suspension was mixed at a 1:1 v/v ratio with 1% alginate (FMC Biopolymer) in PBS, to form a solution of 0.5% alginate and 40, 60, or 100 × 10^6^ cells/ml.

#### Bead constructs

The cell-alginate mixture was dripped directly into a calcium chloride bath (CaCl_2_ solution; 100 mM CaCl_2_ in DDW with 10 mM HEPES buffer), using a syringe without needle, to form ~2–3-mm-diameter beads. The cell-loaded beads were incubated for 15 min at room temperature and then washed 3 times, for 5 min, in PBS (+Ca, +Mg).

#### Plug constructs

Whatman® paper 1 was placed in the bottom of a sterile petri dish after saturating it with CaCl_2_ solution. Teflon molds (6 mm inner diameter, 1-mm thick) were then placed on the CaCl_2_-saturated Whatman paper. The cell-alginate mixture (30 µl) was then pipetted into each mold, and CaCl_2_ solution was gently sprayed on the upper surface. Next, CaCl_2_ solution was added to cover the entire plug and samples were incubated for 15 min at room temperature. After 15 min, the hydrogels were separated from the molds and washed in a PBS (+Ca, +Mg) bath 3 times, for 5 min each.

Beads/plugs were gently transferred to a 24-well plate with BMSC medium and incubated (37 °C, 5% CO_2_, 48 h), after which, the medium was changed.

#### BMSC-seeded collagen matrix

BMSCs were cultured until 80–85% confluent, detached with trypsin-EDTA, and suspended in collagen solution as follows: Type-1 collagen solution (8.44 mg/mL, Corning) was neutralized by mixing 1:1 (v/v) with sterile reconstitution buffer (3 volume of 0.1 N NaOH, 0.07 volume of 5% w/v NaHCO_3_, 1 volume of 100 mM HEPES buffer, and 1 volume of 10× M199) on ice. The cell pellet was then suspended in the collagen solution, resulting in a final collagen concentration of 5 mg/ml and a cell density of 40 × 10^6^ cells/ml. The BMSC-collagen mixture was poured into culture dishes and polymerized at 37 °C for 30 min, after which BMSC medium was placed over the gel and incubated overnight.

### Cell culture, adipogenic differentiation, and maturation

For 2D culture, BMSCs were seeded at a density of 5 × 10^3^ cells/cm^2^ on Matrigel (Cultrex)-coated 6-well plates in BMSC medium. After reaching 80% of confluency (within 24–48 h), the medium was changed to adipogenic differentiation medium (ADM; basal medium supplemented with Rock-inhibitor, WNT inhibitor and fibroblast growth factor). Non-induced BMSCs, cultured in standard medium (BMSC medium), were used as a negative control. Media were changed every other day for 2–3 weeks (the exact period mentioned for each experiment). For the differentiation of BMSC seeded within 3D constructs, the constructs were placed in non-TC, 24-well plates (1 construct per well) and cultured in BMSC medium immediately after seeding (1 ml medium per well). The medium was changed to ADM 3 days post-seeding, allowing the cells to adjust the 3D environment within the constructs before differentiation induction. ADM was changed every other day for 2–3 weeks (the exact periods mentioned for each experiment). For the adipocyte maturation phase, adipogenic maturation medium (AMM; BMSC medium supplemented with insulin and a cocktail of non-animal free fatty acids (FFAs)) based on previous work^9^, was added at different time points following BMSCs differentiation to adipocytes (the exact schedules detailed for each experiment in the “Results” section). The culture handling was performed as described in the differentiation protocol.

### Marble-like construct

Marble-like adipose-muscle tissue was generated using mold casting and 3D scaffold printing methods. In both methods, mature engineered tissues were combined, after culturing each tissue separately.

### Mold-cast marble-like construct

The mature bovine muscle tissue was created as we have previously described^43,44^. Briefly, BSCs were seeded on freeze-dried alginate-RGD scaffolds, incubated in proliferation medium for 1 week and then in myogenic differentiation medium for another 1 week^43^. The BMSC-loaded alginate plugs were adipogenically differentiated for 3 weeks to engineer the bovine adipose tissue. To combine the mature tissues seeded within an alginate scaffold, a previously published protocol was followed^45^. First, the tissues were cut, using a scalpel or a puncher, which exposed the ionic bonds. Two forms of cutting were performed: cutting in half and cutting in a ring-shaped manner. Then, a chelation solution (100 mM sodium citrate and 30 mM EDTA) was pipetted into the cutting area for 10 s (to locally chelate the Ca^2+^ ions from the exposed bonds). The two sections of the different tissues were then attached to each other and re-immersed in CaCl_2_ solution for 10 min, thereby re-cross-linking the alginate. To support integration of the different tissues, the marble-like constructs were cultured in adipogenic maturation medium for 1 week.

### Marble-like construct on 3D-printed scaffolds

#### Extraction of mature adipocytes from the alginate construct

BMSCs were seeded in alginate hydrogel and were differentiated into mature adipocytes, as described above. After adipocyte maturation within the hydrogel, the alginate hydrogel was dissolved, by adding 50 µl of 55 mM sodium citrate in 10 mM HEPES buffer and gently shaking. Upon dissolution, the cells were centrifuged (100 × *g*, 3 min) and the supernatant, containing the mature adipocytes, was gently transferred to a new tube, and mixed 1:1 (v/v) with 1% alginate solution.

#### Fabrication of the muscle tissue on a printed scaffold

The 3D-printed construct was fabricated as previously published^44^. Breifly, ink solution composed of alginate-RGD (1% w/v) and pea protein (1% w/v) was 3D-printed in longitudinal fiber orientation, using a micro-particles-based support bath that was contained 10 mM CaCl_2_ for cross-linking of the printed ink. Next, the 3D-printed scaffold was freeze-dried and then BSCs were seeded onto the scaffold. The cells were allowed to proliferate for 1 week and myogenically differentiate for 1 week^43,44^.

#### Creation of the marble-like construct

In the next step, the mature adipocytes in alginate solution were cast on the 3D-printed scaffold that was covered with differentiated BSCs, and re-cross-linked using 100 mM CaCl_2_ solution, forming an integrated combined construct.

### Oil Red O staining

Prior to staining, cultures were rinsed twice with PBS and fixed with 4% (w/v) paraformaldehyde for 15 min. Oil Red O (ORO) working solution was prepared by diluting 3:2 ORO stock solution (0.5% (w/v) in isopropanol, Sigma) with distilled water and filtering it through a 0.22 µm filter. Cells were incubated with isopropanol (60%) for 5 min and then covered with ORO working solution for 15 min. Cells were then washed with PBS 3–5 times, and then immersed in PBS until analysis. Cells in 3D constructs were fixated in 4% paraformaldehyde (w/v) for 15 min, and then incubated in a 30% (w/v) sucrose solution overnight, embedded in optimal cutting temperature compound (Tissue-Tek), and frozen for subsequent cryo-sectioning (10–20 µm). Samples were imaged under a light microscope (Axio Observer 7 microscope, Zeiss). Images were processed using the Zen Blue software (Zeiss).

### Immunofluorescence (IF) staining of cryo-sections

Cryo-sections were treated with 0.3% Triton X-100 (Bio Lab Ltd.) in PBS (PBS-T) for 5 min, to permeabilize the cell membranes. The sections were then incubated in blocking solution (1% bovine serum albumin (BSA) in PBS-T) for 1 h, at room temperature. Next, sections were incubated with rabbit polyclonal anti-peroxisome proliferator-activated receptor gamma (PPAR-γ) antibody (1:200, Abcam) in PBS-T with 1% BSA, overnight, at 4 °C. After extensive washings with PBS-T 4–5 times, for 5 min each, the sections were incubated with Alexa Fluor 647 goat anti-rabbit IgG (1:400; Invitrogen) and DAPI (1:1000, Sigma) diluted in PBS-T with 1% BSA, for 2 h, at room temperature. Sections were then rinsed 4–5 times with PBS, for 5 min each, and then mounted with Fluromount-G (Southern Biotechnology) and covered with cover slips (#1.5). The slides were then imaged using a fluorescence microscope (Axio Observer 7 microscope, Zeiss).

### Lipid droplet and IF staining of whole-mount scaffolds

Whole scaffolds were fixated with 4% paraformaldehyde (w/v) + 1 mM calcium for 10 min, and then washed several times with PBS containing calcium (PBS-Ca^++^). Next, permeabilization and blocking steps were carried out: the scaffolds were incubated with 0.05% w/v saponin in PBS-Ca^++^ containing 0.2% w/v BSA (S-B-P), for 20 min. Then, the constructs were incubated with mouse monoclonal anti-CEBP antibody (1:200, Abcam) diluted in S-B-P, overnight, at 4 °C. Constructs were then washed with PBS-Ca^++^, 4–5 times, 10 min each, and then were incubated with Alexa Fluor 647 donkey anti-mouse antibody (1:100, Jackson) and DAPI (1:1000, Sigma) in S-B-P, at room temperature, for 1.5 h. Thereafter, HCS LipidTOX™ Green (1:200; Invitrogen) was added to the secondary antibody solution and scaffolds were incubated in the staining solution for another 0.5 h. Then, scaffolds were washed 4–5 times with PBS-Ca^++^, 10 min each, and imaged using a confocal microscope LSM 700 (Zeiss). The marble-like constructs were stained in the same manner except that different primary and secondary antibodies were used—goat anti-desmin (1:100, Santa Cruz Biotechnology) and Alexa Fluor 546 donkey anti-goat (1:400, Life Technologies) or Cy5 donkey anti-goat (1:100, Jackson) antibody, respectively.

Images were processed in ImageJ software for enumeration, area measurement, and size distribution analysis of cellular lipid droplets. All images were taken under the same acquisition conditions. Therefore, the same parameters were used for calculating the surface areas. Lipid droplet diameters were calculated from the measured areas and imported into Excel software to generate a size distribution plot. Lipid droplet coverage was calculated by the total area of lipid droplets divided by the plug area, multiplied by 100%. In the experiment involving 6-week differentiation/maturation periods (Fig. 3a–e), samples (*n* = 4) were imaged in bright field mode at each time point and also fluorescently stained for lipid droplets (LipidTox) and for nuclei (DAPI). The lipid droplets were identified from the bright field images and their area was measured using ImageJ software (Fig. 3c). In the experiment involving 2-week differentiation/maturation periods (Fig. 3f–i), each sample was fluorescently stained for lipid droplets (LipidTox) and for nuclei (DAPI or Draq5) and imaged using a LSM microscope. The lipid droplets were identified from the fluorescent images using the analyze particles tool in ImageJ software (Fig. 3h). Differentiation percentage was calculated by dividing the number of lipid-laden cells by the total number of cells in each construct. The mean lipid droplet content per cell was calculated by dividing the total lipid area by the total number of lipid-laden cells.

### Measurement of mechanical properties

#### Stiffness measurements

Compression testing of BMSC-loaded hydrogels (alginate or collagen) after adipogenic differentiation was performed using an AR-G2 rheometer (TA Instruments, New Castle, DE, USA) equipped with parallel-plate geometry. Stress–strain curves were generated. The cross-sectional area of each sample was measured immediately before performing the compression test. Samples were imaged using a light microscope and the cross-sectional areas were measured using imageJ software. The compressive Young’s modulus, given by the slope of the stress–strain curve in the linear region, was used as the stiffness parameter.

#### Shrinkage measurements

Dimensions of BMSC-loaded hydrogels (alginate or collagen) were monitored by imaging the constructs and measuring their area on the day of seeding, and after 7, 14, and 21 days of differentiation. Shrinkage percentage was calculated by Eq. (1).

1 $$\% \,{{{{{{\rm{shrinkage}}}}}}}=\frac{{{{{{{\rm{area}}}}}}}\,{{{{{{\rm{on}}}}}}}\,{{{{{{\rm{seeding}}}}}}}\,{{{{{{\rm{day}}}}}}}-{{{{{{\rm{area}}}}}}}\,{{{{{{\rm{on}}}}}}}\,{{{{{{\rm{day}}}}}}}\,X}{{{{{{{\rm{area}}}}}}}\,{{{{{{\rm{on}}}}}}}\,{{{{{{\rm{seeding}}}}}}}\,{{{{{{\rm{day}}}}}}}}\;\cdot \;100 \%$$

### Total RNA extraction, cDNA synthesis, and RT-PCR analysis

Total RNA of BMSCs was isolated using the RNeasy Mini Kit (Qiagen) according to the manufacturer’s instructions. Briefly, BMSCs were harvested from culture plates by trypsinization and washed twice with PBS. Then, cell pellets were disrupted by adding RLT buffer plus β-mercaptoethanol and the lysates were homogenized. Next, samples were centrifuged, and supernatants were extracted into a new tube containing ethanol. Samples were then transferred into a RNeasy spin column. The RNA concentration of the final elute was measured using a NanoDrop (Thermo Fisher Scientific) and stored at −80 °C. The RNA samples were reverse-transcribed into complementary DNA (cDNA) using the High-Capacity cDNA Reverse Transcription Kit (ABI; 4374966), according to the manufacturer’s instructions. The cDNA obtained was stored at 4 °C until use in PCR amplification reactions. The PCR primers and the length of the amplified products were as follows: CD29 (GACACGCAAGAAAATCCGAT and ACCGGCAATTTAGAGACCA, 89 bp), CD44 (CGGACCTGCCCAATGCCTTTGA and TGCACAGTTGGGAGGTGCGT, 226 bp), CD73 (TTCTCAACAGCAGCATCCCA and CAGTGCCATCCAGATAGACA, 122 bp), CD105 (CCATCAAAAGCCTGACCTTCGG and AGTCTGATGACCACCTCGTT, 138 bp), negative control CD45 (AAGCTGCGCAGGAGGGTAAACG and AAGCTGCGCAGGAGGGTAAACG, 206 bp), and house-keeping 18S (GGAGCGATTTGTCTGGGTTA and GTAGGGTAGGCACACGCTGA, 214 bp). Amplification was performed using the DreamTaq Hot Start Green PCR Master Mix (Thermo Fisher Scientific, USA), according to the manufacturer’s protocol. The PCR program included an initial hot start at 95 °C for 3 min, followed by 30 cycles of denaturation at 95 °C for 30 s, annealing at 51–60 °C, depending on the primer pair, for 45 s, elongation at 72 °C for 1 min, and ended with the final extension at 72 °C for 5 min. After the amplification reaction, the resulting products were separated by electrophoresis in 1.2% agarose gels and photographed with a UV trans-illuminator.

### Real-time quantitative PCR

RT-qPCR was carried out using TaqMan Fast Universal PCR Master Mix (2×) (ABI; 4352042). The following primers were used: 18S (Hs03003631; Thermo Fisher Scientific), GAPDH (Bt03217547_m1; Thermo Fisher Scientific), and PPARG (Bt03217547_m1; Thermo Fisher Scientific). A total of 10 ng cDNA was used for each reaction, according to the manufacturer’s instructions. The reaction was performed using the QuantStudio Real-Time PCR System (ThermoFisher Scientific, Waltham, USA). The amplification reaction conditions: 95 °C for 20 s, 40 cycles at 95 °C for 1 s, 60 °C for 20 s, in a 10 μL reaction volume. Each sample was processed in three replicates, with 18S/GAPDH used as a reference gene. The ΔΔCT_t_ method was used to determine relative expression levels, where the target gene was normalized to 18S/GAPDH expression. Data were analyzed using QuantStudio design and analysis software version 1.5.1.

### Statistics and reproducibility

All statistical analyses in this study were performed using GraphPad PRISM software. For comparison between two groups only (Figs. 1m, 2c, d and 3e) a two-tailed Student’s unpaired *t*-test was used. For comparison between two groups only at different time points (Fig. 3b) a multiple unpaired *t*-test followed by Holm-Sidak post hoc test was performed. For comparison between multiple groups during a single time point (Figs. 3h and 5b) a one-way ANOVA followed by Tukey’s post hoc test was used. For comparison between multiple groups during several time points (Fig. 2b) a two-way ANOVA followed by Tukey’s post hoc test was performed. Significance was taken at *p* ≤ 0.05 (*), *p* ≤ 0.01 (**), *p* ≤ 0.001 (***), and *p* ≤ 0.0001 (****). Graphs were generated using Excel or GraphPad PRISM software and display all data points and/or mean ± standard error of mean (SEM) including the sample sizes as indicated in the legend of each figure.

### Reporting summary

Further information on research design is available in the Nature Research Reporting Summary linked to this article.

## Supplementary information

Supplementary information

Description of Additional Supplementary Files

Supplementary Data 1

Reporting Summary

## Data Availability

The source data for figures [1, 2, 3 and 5] are provided with the paper (Supplementary Data 1).
