# Supplementary material for: Engineered marble-like bovine fat tissue for cultured meat
Source: Commun Biol. 2022 Sep 8;5:927. doi: 10.1038/s42003-022-03852-5 (PMC9452530; doi:10.1038/s42003-022-03852-5)
Supplement: Supplementary file 1 — Supplementary information [file 42003_2022_3852_MOESM1_ESM.pdf]

Supplementary Figures

Figure S1:

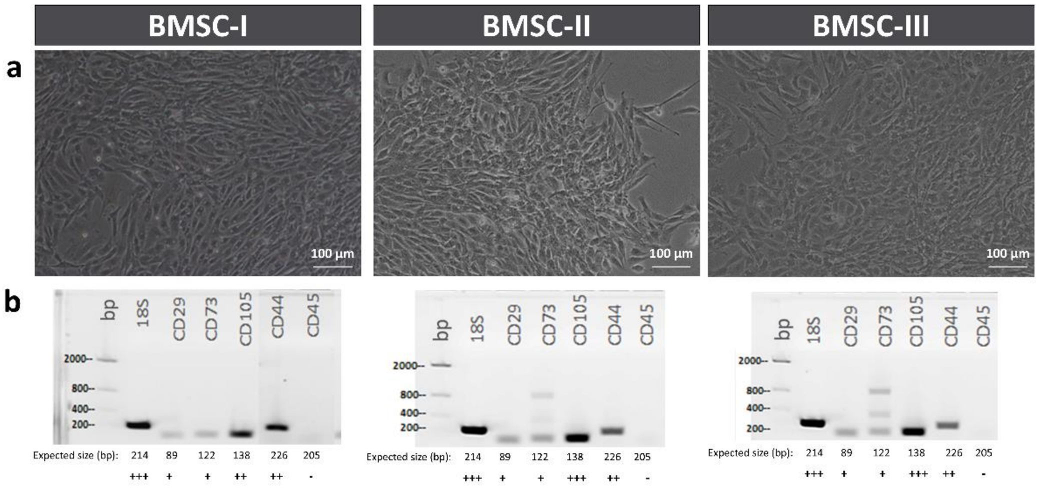

Figure S1: **Bovine adipose-derived MSCs isolation.** (a) The morphology of MSCs derived from bovine peri-renal adipose tissue of three independent isolations (BMSC-I, BMSC-II and BMSC-III) after 5 days of cultivation (passage 0). (b) Gene expression of MSCs markers: RT-PCR analysis of three independent isolations of adipose-derived BMSCs, at passage 2. 18S served as an internal house-keeping gene.

Figure S2:

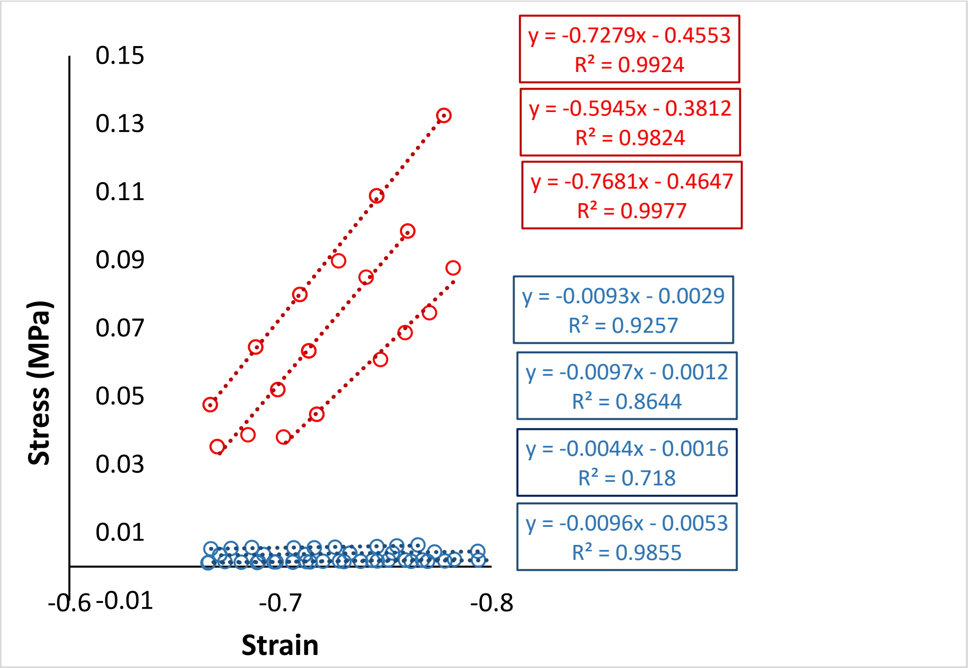

Figure S2: Compressive stress-strain curves: Linear regions of stress vs. strain compression curves and their corresponding linear regression trendlines, linear equations and R-squared of BMSC-loaded alginate (blue) vs. BMSC-loaded collagen (red) after 21 days in differentiation medium.

Figure S3:

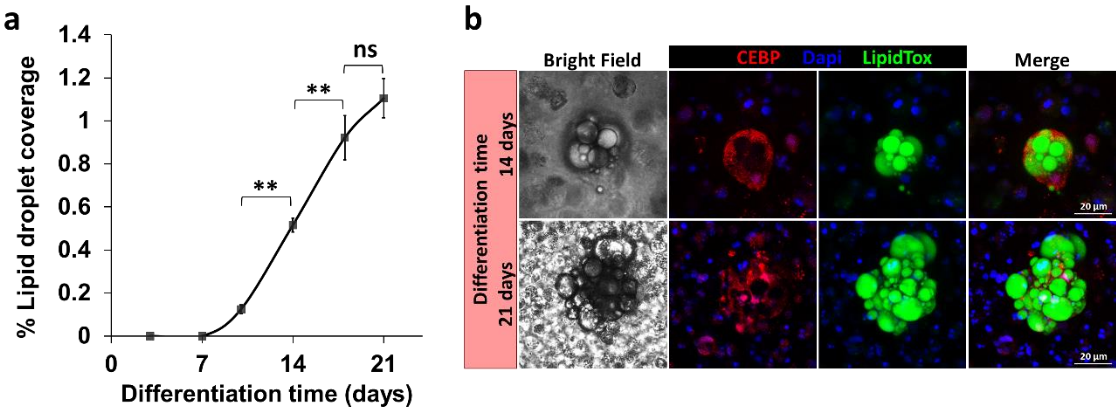

Figure S3: Adipogenic differentiation of BMSC-loaded alginate 3D constructs (a) Lipid droplet coverage profile throughout 21 days of differentiation, n=4, one-way ANOVA followed by Tukey's post-hoc test, $^{**}p<0.01$, ns- non-significant. (b) Whole-mount C/EBPα and lipid staining (red, C/EBPα; green, LipidTox; blue, DAPI) 14- and 21-days post-differentiation.

Figure S4:

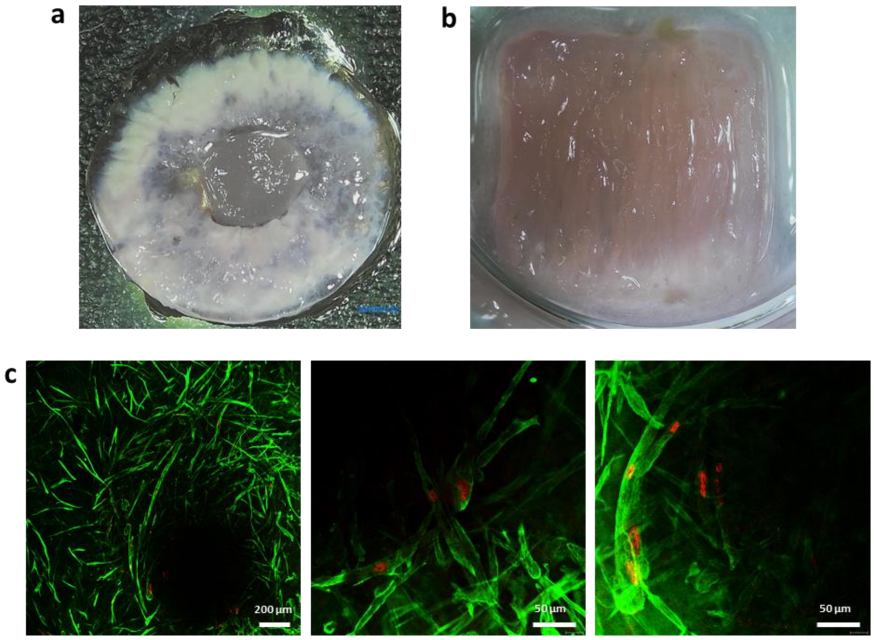

Figure S4: Engineered bovine adipose-muscle marbled- like construct images taken by a camera of the marbled- like constructs of Figure 4. (a) Mold-cast marbled- like constructs composed of fat and muscle in a ring shape. (b) Marbled- like construct on 3D-printed scaffold composed of extracted mature adipocytes (differentiated BMSCs) within bovine engineered muscle tissue. (c) Confocal LSM images of immunostaining for myogenin (red) and desmin (green) (myogenic markers) of the engineered muscle tissue in the marbled- like construct.
