# Supplementary material for: Engineered marble-like bovine fat tissue for cultured meat
Source: Commun Biol. 2022 Sep 8;5:927. doi: 10.1038/s42003-022-03852-5 (PMC9452530; doi:10.1038/s42003-022-03852-5)
Supplement: Supplementary file 2 — Description of Additional Supplementary Files [file 42003_2022_3852_MOESM2_ESM.pdf]

Description of Additional Supplementary Files

File name: Supplementary Data 1
Description: The source data behind the graphs in the paper (Each sheet is named according to the graph it belongs to).
