# Supplementary material for: Engineered marble-like bovine fat tissue for cultured meat
Source: Commun Biol. 2022 Sep 8;5:927. doi: 10.1038/s42003-022-03852-5 (PMC9452530; doi:10.1038/s42003-022-03852-5)
Supplement: Supplementary file 4 — Reporting Summary [file 42003_2022_3852_MOESM4_ESM.pdf]

nature portfolio

Corresponding author(s): Shulamit Levenberg

Last updated by author(s): Aug 5, 2022

Reporting Summary

Nature Portfolio wishes to improve the reproducibility of the work that we publish. This form provides structure for consistency and transparency in reporting. For further information on Nature Portfolio policies, see our Editorial Policies and the Editorial Policy Checklist.

Statistics

For all statistical analyses, confirm that the following items are present in the figure legend, table legend, main text, or Methods section.

n/a	Confirmed	
☐	☒	The exact sample size (*n*) for each experimental group/condition, given as a discrete number and unit of measurement
☐	☒	A statement on whether measurements were taken from distinct samples or whether the same sample was measured repeatedly
☐	☒	The statistical test(s) used AND whether they are one- or two-sided<br>*Only common tests should be described solely by name; describe more complex techniques in the Methods section.*
☒	☐	A description of all covariates tested
☐	☒	A description of any assumptions or corrections, such as tests of normality and adjustment for multiple comparisons
☐	☒	A full description of the statistical parameters including central tendency (e.g. means) or other basic estimates (e.g. regression coefficient) AND variation (e.g. standard deviation) or associated estimates of uncertainty (e.g. confidence intervals)
☒	☐	For null hypothesis testing, the test statistic (e.g. *F*, *t*, *r*) with confidence intervals, effect sizes, degrees of freedom and *P* value noted<br>*Give P values as exact values whenever suitable.*
☒	☐	For Bayesian analysis, information on the choice of priors and Markov chain Monte Carlo settings
☒	☐	For hierarchical and complex designs, identification of the appropriate level for tests and full reporting of outcomes
☒	☐	Estimates of effect sizes (e.g. Cohen's *d*, Pearson's *r*), indicating how they were calculated

Our web collection on statistics for biologists contains articles on many of the points above.

Software and code

Policy information about availability of computer code

Data collection	No software used.
Data analysis	Microscopy images were processed using the Zen Blue and black software (Zeiss). Images were processed in ImageJ and Cellprofiler softwares for enumeration, area measurement and size distribution, Excel software was used for size distribution plots. For all statistic analyses we used GraphPad Prism or Excel. Gene expression data were analyzed using QuantStudio design and analysis software version 1.5.1.

For manuscripts utilizing custom algorithms or software that are central to the research but not yet described in published literature, software must be made available to editors and reviewers. We strongly encourage code deposition in a community repository (e.g. GitHub). See the Nature Portfolio guidelines for submitting code & software for further information.

Data

Policy information about availability of data

All manuscripts must include a data availability statement. This statement should provide the following information, where applicable:

- Accession codes, unique identifiers, or web links for publicly available datasets
- A description of any restrictions on data availability
- For clinical datasets or third party data, please ensure that the statement adheres to our policy

All raw-data is available in the 'Supplementary Data 1' file

Field-specific reporting

Please select the one below that is the best fit for your research. If you are not sure, read the appropriate sections before making your selection.

☒ Life sciences ☐ Behavioural & social sciences ☐ Ecological, evolutionary & environmental sciences

For a reference copy of the document with all sections, see nature.com/documents/nr-reporting-summary-flat.pdf

Life sciences study design

All studies must disclose on these points even when the disclosure is negative.

Sample size	The sample size was equal or larger than 3 for all experiments. No method was used to determine the sample size.
Data exclusions	No data were excluded.
Replication	replicate experiments were successful and gave similar results.
Randomization	Samples were randomly allocated into experimental groups, randomized during seeding, media change and analyses.
Blinding	For gene expression assays and for microscopy imaging samples were given serial numbers to prevent human bias.

Reporting for specific materials, systems and methods

We require information from authors about some types of materials, experimental systems and methods used in many studies. Here, indicate whether each material, system or method listed is relevant to your study. If you are not sure if a list item applies to your research, read the appropriate section before selecting a response.

Materials & experimental systems

n/a	Involved in the study	
☐	☒	Antibodies
☒	☐	Eukaryotic cell lines
☒	☐	Palaeontology and archaeology
☒	☐	Animals and other organisms
☒	☐	Human research participants
☒	☐	Clinical data
☒	☐	Dual use research of concern

Methods

n/a	Involved in the study	
☒	☐	ChIP-seq
☒	☐	Flow cytometry
☒	☐	MRI-based neuroimaging

Antibodies

Antibodies used

Rabbit polyclonal anti-PPAR-γ antibody (ab45036, AbCam)
Mouse monoclonal Anti-CEBP Alpha/CEBPA antibody [15C8] (ab15047, AbCam)
Goat α-desmin (Y-20) (sc-7559; lot#52516; Santa Cruz Biotechnology)
Alexa Fluor 647 goat anti-rabbit IgG (Invitrogen)
Alexa Fluor 647 donkey α-mouse IgG (715-605-151, Jackson)
Alexa Fluor 546 donkey α-goat (Life technologie)
Cy5 donkey anti-goat (Jackson)

Validation

All antibodies were validated by the respective manufacturers.
